# Enhancing Florida red tilapia aquaculture: biofloc optimization improves water quality, pathogen bacterial control, fish health, immune response, and organ histopathology across varied groundwater salinities

**DOI:** 10.1007/s11259-024-10433-w

**Published:** 2024-07-03

**Authors:** Mohamed M. Abdel-Rahim, Ashraf. I. G. Elhetawy, Wael A. Shawky, Samy Y. El-Zaeem, Alaa A. El-Dahhar

**Affiliations:** 1 Aquaculture Division, National Institute of Oceanography and Fisheries, NIOF, Cairo, Egypt; 2 Animal and Fish Production Department, Faculty of Agriculture, Alexandria University, Saba-basha, Egypt

**Keywords:** Groundwater, Biofloc, Florida red tilapia, Different salinities, Fish performance and well-being, Pathogenic bacterial load

## Abstract

Freshwater scarcity poses challenges to aquaculture worldwide, including countries like Egypt. In this study, we investigate the feasibility of integrating underground saline water (USW) with varying salinities into a Biofloc (BFT) system for desert mariculture of Florida red tilapia (FRT) and its impacts on water quality, fish performance and health. Four BFT treatments (C/N ratio = 15) were examined in triplicate using four salinity levels 0 ppt, 12 ppt, 24 ppt and 36 ppt, expressed as S0, S12, S24 and S36, respectively. For 75 days, a total of 12 fiberglass tanks (each 250 L^−1^ water) were used to store FRT fry (average weight of 1.73 ± 0.01 g/fish). The fish were fed an experimental diet (protein/fat = 30/5) and an additional carbon source of rice bran. The results revealed that group S12 showed better growth indicators, higher survival rate, lower FCR, and lower ammonia levels, while group S0 exhibited lower growth indicators (final weight, weight gain, and specific growth rate) than all groups. The serum kidney, liver, and antioxidant indices performed better in the S12 group. At 12 ppt, the immune-related parameter (IgM) increased by 22.5%, while the stress parameter (cortisol) decreased by 40.8% compared to the S0 group. The liver and intestinal histopathological results revealed that the S12 and S24 groups performed better. Pathogenic bacterial load counts favored the S24 group, which had the lowest number among the groups studied. The recommended salinity for FRT cultivation in USW and BFT is 19.94-20 ppt, determined by polynomial regression of FW and FCR.

**Supplementary Information:**

The online version contains supplementary material available at 10.1007/s11259-024-10433-w.

## Introduction

Egypt’s groundwater is one of the largest water resources, coming after the Nile River as a second source (Negm and Elkhouly 2021), and therefore plays an important role in economic growth and sustainable development (El-Rawy et al. 2019). The total reserve of groundwater reservoirs in Egypt is projected to be around 1200 billion m^3^ with varying degrees of salinity, most of which is brackish and saline water (Salim 2012; Geriesh et al. 2023). Consequently, and given the severe problems associated with freshwater scarcity, there is a major trend to maximize the effective use of accessible groundwater and expand desert mariculture as a promising aquaculture sector (Lotfy et al. 2021, 2023; Abdel-Rahim et al. 2023).

Recently, the aquaculture strip has grown tremendously due to the diversity of farming systems and techniques; it now supplies around half of the world’s animal protein needs, protects endangered species, and allows fish breeders to cultivate fish outside of their native habitat (Elhetawy et al. 2023a). In 2020, the world’s aquaculture production reached 122.6 million tons, equivalent to 281.5 billion US dollars (FAO 2022; Lotfy et al. 2023). Tilapia fish is an important fish species for aquaculture on a global scale due to its fast growth rate, tasty flesh, and ease of reproduction in captivity. Additionally, they are great in aquaculture since they can withstand a lot of stress (Nassar et al. 2021). With a production capacity of 1,591,900 tons, Egypt’s aquaculture ranks sixth internationally (Elhetawy et al. 2023a). It is the largest in Africa, accounting for 67.62% of Africa’s contribution to global aquaculture production, which reached 2,354,300 tons in 2020 (FAO 2022; Lotfy et al. 2023). In 2020, Egypt came third globally in tilapia production, contributing a total of 954,154 tons to the global cosmopolitan production of 4,407,200 tons. (FAO 2022; Geletu and Zhao 2023).

Globally, there has been a significant increase in red tilapia aquaculture production in several countries, including Thailand, Malaysia, and China (Jayaprasad et al. 2011; Osman et al. 2023). Genetic variations in red tilapia are commonly believed to have originated from crossing blue tilapia (*O. aureus*) and Nile tilapia (*O. niloticus*) with mutant reddish orange Mozambique tilapia (*Oreochromis mossambicus*) (Reich et al. 1990; Romana-Eguia and Eguia 1999). Red tilapia fish are considered one of the most significant varieties of farmed fish worldwide, due to a variety of beneficial characteristics not found in most other farmed species, particularly tolerance to a wide range of salinity, fast growth rate, adaptation to most cultural systems, the absence of a black membrane in their body cavity, and higher market price compared to other black tilapia species (Pradeep et al. 2014; Aly et al. 2017; Nassar et al. 2021). By mating a female Wami tilapia (*Oreochromis hornorum)* with a male Mozambique tilapia, the red-gold strain of tilapia known as Florida red tilapia (FRT) was developed in the 1970s (Behrends et al. 1982). Egypt received this strain in 1993 (Aly et al. 2017). Salinity levels above 20 ppt kill Nile tilapia, although the more tolerant strain of FRT (*O mosambicus* × *O. hornorum)* can grow in waters with salinity levels as high as 36.2% (Watanabe et al. 2006), with an ideal limit of 17.8% (El-Sayed 2006). Therefore, red tilapia is the most tolerant species of tilapia when it comes to hypersaline water and thus grows widely across the world in various culture systems including the biofloc system (BFT).

BFT is an active aquaculture production technique that requires minimal water exchange to function (Sgnaulin et al. 2018). BFT is one of the sustainable aquaculture systems that is based on the nutrient recycling principle with the addition of a carbon source to maintain a proper C/N ratio and provide predominance to heterotrophic microorganisms (Rind et al. 2023). Microbe aggregates are the backbone of the biofloc system; they aid in controlling the accumulation of harmful nitrogen compounds, supply naturally occurring living food in the form of floc particles, and improve disease resistance (Avnimelech 2009; Habib et al. 2024; Tasleem et al. 2024). Maintaining a balanced carbon to nitrogen ratio and ensuring constant aeration and agitation in the water are mandatory for continuous microbial activity (Soliman and Abdel-Tawwab 2022; Tasleem et al. 2024). Furthermore, the high nutritional value of biofloc means that farmed species consume less artificial feed while receiving a higher microbial protein content. (Zaki et al. 2020; Rind et al. 2023). Therefore, the integration of BFT and underground saline water (USW) may improve the water parameters, allowing fish to exhibit better growth and health, and thus allowing the expansion of desert mariculture.

Literature on the effect of salinity on the performance and health status of red tilapia under BFT conditions is limited (Kumari et al. 2021; Osman et al. 2023). Water salinity can interfere with fish homeostasis and impact their performance (Moorman et al. 2015). Although fish adapt to variations in salinity, it may be necessary for them to expend more energy to maintain isosmotic equilibrium (Rahmah et al. 2020). If the environmental salinity level is significantly different from the isosmotic salinity level, the additional energy required for osmoregulation will have an impact on the protein requirements of red tilapia, which in turn affects their performance (Osman et al. 2023). Given the large quantities of brackish groundwater, most of which falls within the salinity range appropriate for the rearing of red tilapia, its use represents a major incentive for the development of desert aquaculture, leading to a decline in the use of freshwater in aquaculture, expanding mariculture, employing labor, increasing national income, etc. To our knowledge, no research has been undertaken to investigate the impact of different salinity levels in USW on FRT performance in a desert BFT culture. This study investigates the effects of different salinity levels mimicking desert groundwater on the growth, feed utilization, and physiological responses of FRT raised in a BFT system.

## Materials and methods

### Experimental location and water sources

This experiment was carried out at El-Max Research Station, National Institute of Oceanography and Fisheries (NIOF), Alexandria, Egypt. In this trial, two sources of water were used, including USW and 100% dechlorinated freshwater supplied by the municipality with a salinity of 0.27 ppt. The chemical analysis of the USW is as follows: salinity 36.2 ± 0.1 ppt, pH 8.06 ± 0.1, total ammonia nitrogen (TAN) 0.41 ± 0.035 mg L^−1^, manganese 85.2 ± 1.08 µgL^−1^, iron 99.3 ± 2.2 µgL^−1^, copper 5.3 ± 0.005 µg L^−1^, zinc 6.5 ± 0.002 µg L^−1^, cadmium 40.0 ± 1.0 µg L^−1^, chrome 66.0 ± 2.0 µg L^−1^, cobalt 50.0 ± 2.0 µg L^−1^, nickel 70.0 ± 5.0 µg L^−1^, lead 28.0 ± 3.0 µg L^−1^, and total hardness 5823.7 ± 12.2 mg L^−1^. Salinity levels of 12 ppt and 24 ppt were obtained by mixing fresh water and USW.

### Methodology of biofloc initiation and development

Bioflocs were created utilizing Avnimelech (2007); (2009) techniques in three 2 m^3^ fiberglass jars filled with dechlorinated water and a 15: 1 carbon: nitrogen (C: N) ratio. The biofloc was created using an initial sample of freshwater collected from tilapia ponds. To produce biofloc biomass, a carbohydrate source (rice bran) was added to the experimental tanks at a C: N ratio of 15:1. The chemical composition of rice bran was used to determine the amount needed (Table 1). To increase biofloc growth and development, the carbohydrate source was weighed, placed in 5-liter plastic containers, thoroughly mixed with the experimental tanks’ water, and evenly distributed around the tanks’ surface.

**Table 1:** Formulation and proximate compositions of the experimental diet (% on DM basis) and rice bran used for feeding Florida red tilapia reared in a BFT culture system using underground water with different salinities

Ingredients		(g/kg)
Soybean (43% CP)		240
Wheat bran		150
Shrimp meal		125
Rice bran (defat)		106
Wheat bran		100
Fish meal (62% CP)		90
Corn (7.5% CP)		80
Corn gluten		60
Bunder (gelatin)		10
Dicalcium Phosphate		10
Fish oil		8
Soybean oil		8
Trace mineral premix^a^		5
Vitamin premix^a^		5
Antitoxins (Bentonite)^b^		2
Vitamin C		1
Total		1000
Chemical compositions (%)		
Parameters (%)	Experimental diet	Rice bran
Dry matter	92.55	89
Crude protein	30.17	8.1
Ether extract	5.26	12
Crude fiber	6.94	18.8
Ash	11.30	10
NFE^c^	38.88	40.1
Gross energy (MJ/kg)^d^	15.89	13.55
Gross energy (kcal/kg)^e^	3795.26	3236.36

^a^Trace mineral premix and vitamin premix were purchased from Trouw Nutrition Egypt. (https://www.trouwnutrition.com/en/about-us/global-presence/); ^b^Bentonite (Abdel-Rahim et al. 2023); ^c^NFE is nitrogen free extract, NFE% = (100– (moisture % + Crude protein % + lipids % + fibers % + ash %); ^d^Gross energy, MJ/kg was calculated using a value of 23.6 KJ/g proteins, 39.5 KJ/g fat, and 17.2 KJ/g carbohydrates (NFE); ^e^  Megajoule/Kilogram = 238.8458966 Kilocalorie/Kilogram

### Experimental design, fish and feeding regime

Four treatments were administered in triplicate using four salinity levels, as follows: salinity level 0 ppt; salinity level 12ppt; salinity level 24 ppt and salinity level 36 ppt, abbreviated S0, S12, S24 and S36, respectively. Groups S12 and S24 used a mixture of dechlorinated freshwater and USW to reduce salinity to desired levels (S12, S24). The FRT fish were purchased from K21 Marine Fish Hatchery, General Authority for Fisheries Resources Development (GAFRD), Egypt. Before the start of the experiment, the fish were acclimatized to culture water in 2000 L fiberglass tanks and fed a basal diet for three weeks. After acclimatization, a total of 600 apparent healthy FRT fingerlings weighing 1.73 ± 0.01 g were randomly stocked in 12 fiberglass tanks (each 250 L^−1^ water) at a stocking rate of fifty fry per tank. Before stocking fish in the experimental tanks, 100 mL of biofloc per tank (250 L^−1^ water) was added as an initial stock of microbial species of biofloc. For 75 days, the fish were fed an experimental diet containing 30% protein and 5% fat (Table 1) at 10% body weight, and then the feeding rate was gradually reduced to 6% daily at the end (last 15 days) of the experiment. The fish were fed three times a day, seven days a week at 9:00 am, 12:00 pm, and 16:00 pm. Each two weeks, the fish were collected using a soft net, weighed, and then put back in the culture tanks. The amount of feed was adjusted every two weeks based on the actual biomass of the fish. The tanks were bottom- siphoned every two days to eliminate uneaten feed and feces; therefore, the BFT-treated tanks received a small quantity of water to compensate for siphoning and evaporation (up to 0.5% per day). The tanks were continuously aerated using an air blower (3 hp) throughout the experiment, and the fish were kept on a natural light cycle (12 h of light: 12 h of darkness).

### Water quality assessment

Salinity (mS/cm converted to ppt), temperature (° C), pH and dissolved oxygen (ppm) were recorded three times a week, using the SensoDirect 150, a multiparameter portable photometer. TAN, unionized ammonia (NH3), nitrate (NO3), nitrite (NO2), alkalinity, and hardness were measured once a week during the experimental period, as per APHA (2005), using the portable photometer Hanna HI-97,715 Medium Range Ammonia (https://hanna-worldwide.com/; Hanna Instruments, Romania). NH_3_ (ug/L) was calculated using TAN (mg/L), salinity, pH and temperature. The titrimetric method was employed to determine alkalinity (Eaton et al. 2005). Total hardness and nitrate were assessed using the YSI professional plus multiparameter instrument (https://www.ysi.com/proplus). After 30 min of settling, Imhoff cones were used to measure the weekly biofloc volume (FV, mL/L) (Avnimelech and Kochba 2009).

### Growth performance and feed utilization

To assess growth performance and feed utilization indices at the end of the rearing trial, fish were collected, counted and weighed and morphometric measurements of the fish were estimated according to the following equations: (Xu et al. 2016; Ghafarifarsani et al. 2022; Elhetawy et al. 2023b)

$$\mathrm{Weight}\;\mathrm{gain}\left(\mathrm{g}/\mathrm{fish}\right)=\mathrm{final}\;\mathrm{weight}\;\mathrm{-initial}\;\mathrm{weight}.$$

$$\mathrm{Average}\;\mathrm{daily}\;\mathrm{gain},\;\mathrm{ADG}\;(\mathrm g/\mathrm{fish}/\mathrm{day})\;=\;\mathrm{final}\;\mathrm{weight}\;\mathrm{initial}\;\mathrm{weight}/\mathrm{days};$$

$$\mathrm{Specific}\;\mathrm{growth}\;\mathrm{rate}\;(\mathrm{SGR},\;\%/\mathrm{fish}/\mathrm{day})\;=100\;\times\;(\ln\;\mathrm{final}\;\mathrm{weight}-\ln\;\mathrm{initial}\;\mathrm{weight})/\mathrm{days}.$$

$$\mathrm{Survival}\;(\%)\;=\;100\;\times\;(\mathrm{final}\;\mathrm{number}\;\mathrm{of}\;\mathrm{fish}/\mathrm{initial}\;\mathrm{number}\;\mathrm{of}\;\mathrm{fish}).$$

$$\mathrm{Feed}\;\mathrm{conversion}\;\mathrm{ratio}\;(\mathrm{FCR},\;\mathrm g)\;=\;\mathrm{feed}\;\mathrm{consumption}\;(\mathrm g)/\mathrm{weight}\;\mathrm{gain}\;(\mathrm g).$$

$$\mathrm{Protein}\;\mathrm{efficiency}\;\mathrm{ratio}\;(\mathrm{PER},\;\mathrm g)\;=\;\mathrm{total}\;\mathrm{weight}\;\mathrm{gain}\;(\mathrm g)/\mathrm{protein}\;\mathrm{intake}\;(\mathrm g).$$

$$\mathrm{Energy}\;\mathrm{utilization}\;(\mathrm{EU},\;\%)\;=\;100\;\times\;(\mathrm{energy}\;\mathrm{gain}\;(\mathrm{kcal})/\;\mathrm{energy}\;\mathrm{intake}\;(\mathrm{kcal})).$$

$$\mathrm{Protein}\;\mathrm{productive}\;\mathrm{value}\;(\mathrm{PPV}\%)\;=\;100\;\times\;(\mathrm{protein}\;\mathrm{gain}\;(\mathrm g)/\;\mathrm{protein}\;\mathrm{intake}\;(\mathrm g)).$$

Energy gain (Kcal) = energy content in the fish carcass (Kcal) at the end − energy content in the fish carcass (Kcal) at the start.

### The proximal composition of fish, feed and biofloc biomass

The whole-body chemical composition (moisture, crude protein, crude fat, fiber, and ash) of fish (ten fish) at the start and (ten fish per replicate, and thirty fish per group) at the end, as well as experimental feed and biofloc, were analyzed using AOAC (2000). For biofloc analysis, water samples (20 L) from each group treated with BFT were filtered through 20 μm nets. The collected biofloc was then transferred to a Petri dish and dried in an oven at 55 °C for 72 h (Bakhshi et al. 2018; Khanjani et al. 2021).

### Hematological analysis

Blood samples were taken from the caudal vertebral vein of an anesthetized fish (0.25 ml/liter clove oil) at the end of the experiment, with six fish per tank and eighteen per group. The blood sample was used to undergo hematologic examination and was separated by centrifugation of the coagulated blood at 4000 rpm for 15 min at 4 ° C without anticoagulant before being stored at -20 ° C until analysis.

Serum creatinine (CRE) was determined using the colorimetric method (Heinegård and Tiderström 1973). The indicators of kidney function (urea nitrogen, uric acid, and ammonia) were measured according to Whitehead et al. (1991). Serum aspartate aminotransferase (AST) and alanine aminotransferase (ALT) were estimated according to the method described by Bergmeyer et al. (1978). Alkaline phosphatase (ALP) activity was determined using an enzymatic colorimetric method according to Tietz et al. (1983). Total proteins (TP) and albumins (ALB) were determined according to Hedayati et al. (2019) using commercial kits (Bio-diagnostics, Giza, Egypt). Serum globulin (GLO) was determined by subtracting the ALB value from the TP value of the same sample. The methods described by Trinder (1969) were used to assess serum glucose (GLU). Total cholesterol (CHO) was assessed using the method of Allain et al. (1974). The serum triglyceride (TG) level was analyzed using the TG quantification kit (MAK266, Sigma-Aldrich, St Louis, MO, USA). In this assay, TG is converted to free fatty acids and glycerol (Fossati and Prencipe 1982). High-density lipoprotein (HDL) and low-density lipoprotein (LDL) were determined according to the method described by Vassault (1983). The activity of amylase and lipase was assessed according to Zamani et al. (2009). Cortisol (COR) was assessed using the methods described by Foster and Dunn (1974). Serum total immunoglobulin M (IgM) was determined by precipitating Ig with polyethylene glycol and subtracting the initial and final total protein according to Siwicki (1993). Malondialdehyde (MDA) levels were determined according to Uchiyama and Mihara (1978). Catalase (CAT) enzyme was estimated according to the method of Aebi (1984). Using an automatic biochemical analyzer, total antioxidative capacity (TAC) activities were assessed (Hitachi 7600D, Hitachi, Tokyo, Japan) using the method of Koracevic et al. (2001). Glutathione peroxidase (GPx) levels were detected using the method of Paglia and Valentine (1967).

### Histology of the liver and intestine tissues

At the end of the experiment, the livers and intestines (anterior and posterior segments) of four fish per replicate (*n* = 12 fish/each treatment) were collected and immediately fixed in 10% neutral buffered formalin, for 48 h. Subsequently, tissue samples were processed using the paraffin embedding procedure described by Bancroft et al. (2013). Following that, the tissues were dehydrated in ethyl alcohol at successive grades, clarified in xylene, blocked in paraffin wax, cut into several 5-µm-thick sections, and finally stained with hematoxylin and eosin (H & E stain). Next, several representative photomicrographs of the sections produced were recorded using a digital camera (Leica EC3, Leica, Germany) attached to a microscope (Nikon E200, Tokyo, Japan). High-power microscopic fields (HPF) were used to measure intestinal villi length (µm), villi width (µm), inter villi space (µm), intestinal muscular layer thickness (µm), and intestinal goblet cells (#/mm2), in the red tilapia intestine.

### Pathogenic bacterial load

At the end of the experiment, water samples were obtained to count pathogenic microorganisms in the experimental units using pre-specified methods described by PHE (2014), as presented in the Supplementary Information (SI). The pathogenic bacteria investigated in this study are *Streptococcus sp., Vibrio sp., Salmonella sp., Staphylococcus aureus*, *Aeromonas sp.* and *total aerobic count* (Table 8).

### Statistical analyzes

The data were analyzed for normality (Shapiro-Wilk’s test) and homoskedasticity (Levene’s test). Data were presented as means ± SEM (standard error of the mean) using SPSS software V26. The data were analyzed using SPSS one-way ANOVA and Duncan’s test. All differences were statistically significant at *P* < 0.05. Regression analysis was used to determine the reference line (highest response level) for the final weight and feed conversion ratio (FCR).

## Results

### Water quality data and floc volume

**Fig. 1:**
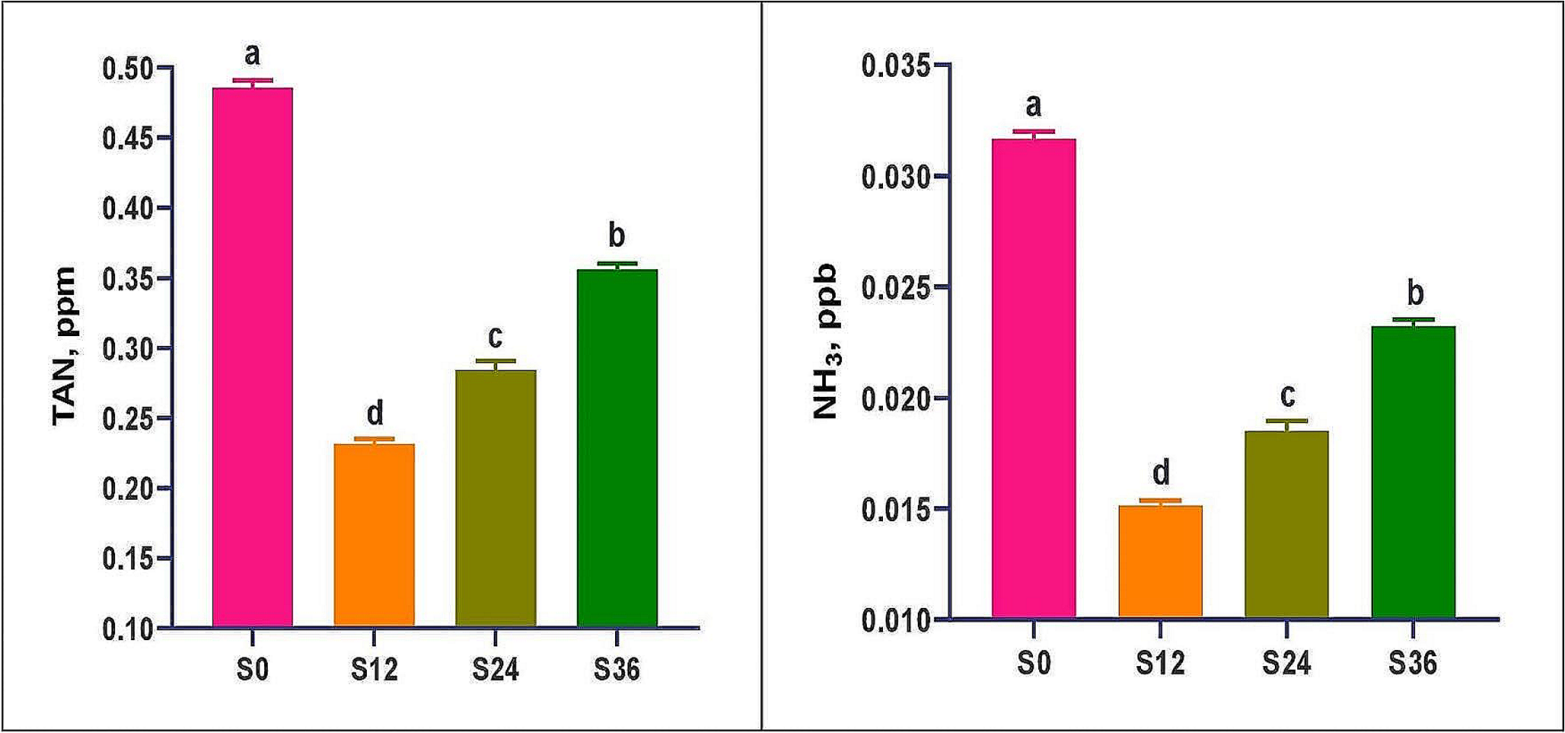
Total ammonia nitrogen (TAN) and un-ionized ammonia (NH3) of Florida red tilapia grown in BFT-based tanks using underground water with different salinities, for 75 days. Where BFT = biofloc; S0 = water with a salinity of 0 ppt (fresh water); S12 = water with a salinity of 12 ppt; S24 = water with a salinity of 24 ppt; and S36 = water with a salinity of 36 ppt

The following parameters were recorded: temperature (27.5 to 27.77 C), pH (7.49 to 8.3), dissolved oxygen (6.53 to 6.88 ppm), total alkalinity (66.0 to 328.2 ppm), NH_3_ (15.13 to 31.67 ppb), nitrate-N (1.57 to 4.07 ppm), nitrite-N (0.31 to 0.46 ppm) and total hardness (67.67 to 5823.67 ppm) (Table 2a). With the exception of temperature, all investigated parameters exhibited significant differences (*P* < 0.05) between treatments. Group S12 had the lowest TAN and NH_3_ values, while S0 had the highest (Fig. 1). The volume of floc (mL / L) differed significantly (*P* < 0.05) between treatments throughout the trial, with the highest volume (36.33) observed in S0 and the lowest (23.20) in S36 (Table 2a). Table 2b (supplementary information) displays the ranges of water quality parameters within each group during the 75-day experimental period.

**Table 2:** Physicochemical parameters of underground water with different salinities used for Florida red tilapia culture in BFT-based tanks during a 75-day experimental period

Parameters	S0	S12	S24	S36	*P* value
Temperature, ^◦^C	27.50 ± 0.10	27.73 ± 0.09	27.67 ± 0.12	27.70 ± 0.10	0.434
pH	7.49 ± 0.03^d^	7.63 ± 0.02^c^	7.78 ± 0.03^b^	8.03 ± 0.04^a^	0.001
DO, ppm	6.88 ± 0.03^a^	6.53 ± 0.03^b^	6.58 ± 0.05^b^	6.58 ± 0.02^b^	0.001
TAN, ppm	0.486 ± 0.005^a^	0.232 ± 0.004^d^	0.284 ± 0.006^c^	0.356 ± 0.004^b^	0.001
NH3, ppb	31.67 ± 0.35^a^	15.13 ± 0.23^d^	18.50 ± 0.46^c^	23.23 ± 0.29^b^	0.001
Nitrate-N, ppm	1.57 ± 0.18^d^	2.57 ± 0.12^c^	3.47 ± 0.03^b^	4.07 ± 0.12^a^	0.001
Nitrite-N, ppm	0.310 ± 0.012^b^	0.337 ± 0.015^b^	0.350 ± 0.012^b^	0.460 ± 0.015^a^	0.001
Alkalinity, ppm	66.0 ± 2.37^d^	116.5 ± 3.90^c^	212.2 ± 4.27^b^	328.2 ± 3.87^a^	0.001
Hardness, ppm	67.67 ± 4.6^d^	2723.67 ± 19.2^c^	3895.33 ± 17.3^b^	5823.67 ± 12.2^a^	0.001
Floc volume, ml/L	36.33 ± 0.35^a^	30.40 ± 0.64^b^	27.73 ± 0.34^c^	23.20 ± 0.25^d^	0.001

Values in the same row with a different superscript are significantly different (*P* ≤ 0.05). Where BFT = biofloc; S0 = water with a salinity of 0 ppt (fresh water); S12 = water with a salinity of 12 ppt; S24 = water with a salinity of 24 ppt; and S36 = water with a salinity of 36 ppt; TAN = Total ammonia nitrogen; and NH3 = un-ionized ammonia

### Growth performance, survival, feed utilization

**Fig. 2:**
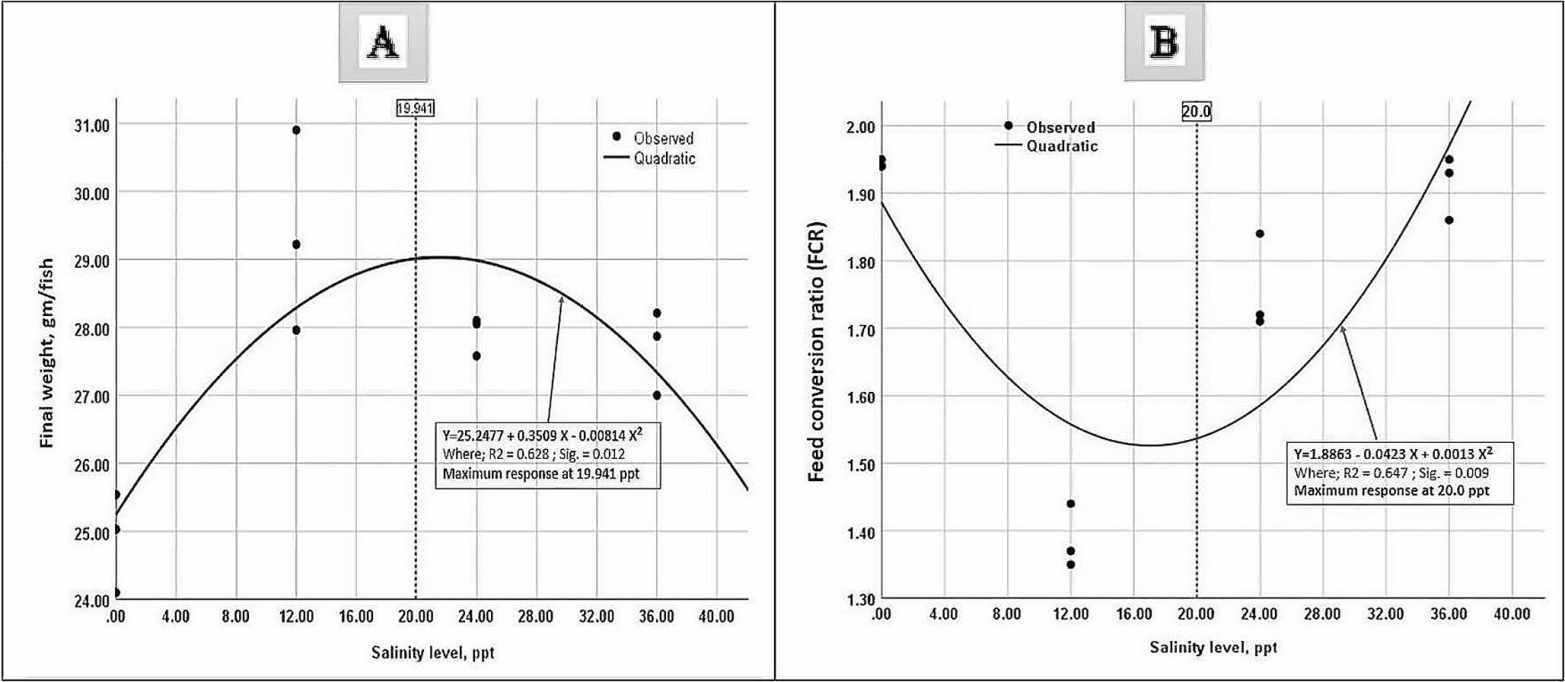
The best salinity level calculated by the quadratic polynomial regression of the final weight and feed conversion ratio (FCR) of Florida red tilapia grown in BFT-based tanks using underground water with different salinities, for 75 days. Where BFT = biofloc; Y = final weight (A) or FCR (B); X = salinity level

Table 3 shows how salinity affects the growth and feed utilization of FRT raised under BFT conditions. Salinity levels (12–36 ppt) had no significant effect on the growth performance of the red tilapia, however there were significant differences (*P* < 0.05) between S0 and the other treatments. The S12 group had the highest growth rate, and the S0 group had the lowest. S12 outperformed S0 in terms of final weight, ADG and SGR, achieving 17.96%, 19.1%, and 5.9%, respectively. The optimal salinity level for the best growth performance was determined by quadratic polynomial regression of the final weight at 19.94 ppt (Fig. 2). The survival rate varied significantly between treatments, with S12 having the highest record (88.67%), but no significant differences were found between the other treatments (S0, S24, and S36). In the same context, salinity levels influenced all feed utilization parameters (Table 3). The S12 treatment had the lowest numerical FCR value (1.39), while the S0 treatment had the highest (1.94). The S12 treatment had the highest significant values of PER, PPV, and energy utilization value, while the lowest values were observed for S0. Quadratic polynomial regression of the final weight at 20.0 ppt revealed the appropriate salinity level for the lowest FCR (Fig. 2).

**Table 3:** Growth performance and feed utilization of Florida red tilapia grown in BFT-based tanks using underground water with different salinities, for 75 days

Parameters/Treatments*		S0	S12	S24	S36	*P* value
Growth performance	Initial weight (gm/fish)	1.73 ± 0.01	1.74 ± 0.01	1.73 ± 0.01	1.73 ± 0.01	0.752
	Final Weight (gm/fish)	24.89 ± 0.42^b^	29.36 ± 0.85^a^	27.91 ± 0.17^a^	27.69 ± 0.36^a^	0.002
	Gain (gm/fish)	23.16 ± 0.42^b^	27.62 ± 0.86^a^	26.18 ± 0.18^a^	25.97 ± 0.36^a^	0.002
	ADG (gm/fish/day)	0.309 ± 0.006^b^	0.368 ± 0.012^a^	0.349 ± 0.002^a^	0.346 ± 0.005^a^	0.002
	SGR (%/fish/day)	3.56 ± 0.02^b^	3.77 ± 0.05^a^	3.71 ± 0.018^a^	3.70 ± 0.017^a^	0.005
Survival, %		80.00 ± 2.00^b^	88.67 ± 2.40^a^	82.00 ± 2.31^b^	78.67 ± 0.67^b^	0.030
Feed utilization	FCR	1.94 ± 0.00^a^	1.39 ± 0.03^c^	1.77 ± 0.04^b^	1.91 ± 0.03^a^	0.001
	PER (gm)	2.07 ± 0.01^c^	2.90 ± 0.06^a^	2.29 ± 0.06^b^	2.10 ± 0.04^c^	0.001
	PPV (%)	35.40 ± 0.77^c^	49.42 ± 0.90^a^	39.44 ± 1.45^b^	34.33 ± 0.64^c^	0.001
	Energy gain (Kcal)	40.85 ± 0.19^c^	48.99 ± 1.65^a^	46.90 ± 0.16^ab^	45.66 ± 0.97^b^	0.002
	Energy utilization (%)	20.85 ± 0.62^c^	29.40 ± 0.81^a^	23.44 ± 0.73^b^	21.08 ± 0.43^c^	0.001

Means within the same row with different superscripts are significantly different (*P* ≤ 0.05). Where BFT = biofloc; S0 = water with a salinity of 0 ppt (fresh water); S12 = water with a salinity of 12 ppt; S24 = water with a salinity of 24 ppt; and S36 = water with a salinity of 36 ppt; ADG = average daily gain; SGR = specific growth rate; FCR = feed conversion ratio; PER = protein efficiency ratio; PPV = protein productive value

### Proximate composition of the carcass of red tilapia and biofloc

The proximate chemical composition of the carcass and biofloc of tilapia is shown in Table 4. There were no statistically significant differences (*P* < 0.05) in dry matter, ash, and carcass energy of FRT, however, there was a significant difference (*P* < 0.05) in crude protein and lipids between treatments (S36 versus other treatments; S0, S12 and S24). In biofloc composition, S36 had the highest percentage of dry matter (29.63 ± 0.06), while S0 had the highest crude protein, ether extract and gross energy values. Biofloc treatments at salinity levels ranging from 12 to 36 ppt exhibited significantly higher NFE values than the S0 group.

**Table 4:** Biofloc and whole-body chemical composition of Florida red tilapia grown in BFT-based tanks using underground water with different salinities, for 75 days

Parameters/Treatments*		S0	S12	S24	S36	*P* value
Carcass composition	Dry matter, %	29.78 ± 0.71	29.85 ± 0.19	30.12 ± 0.39	29.35 ± 0.34	0.694
	Protein, %	57.37 ± 0.22^a^	56.95 ± 0.27^a^	57.01 ± 0.29^a^	55.75 ± 0.46^b^	0.035
	Ether extract, %	28.03 ± 0.09^b^	28.47 ± 0.14^b^	28.46 ± 0.39^b^	29.71 ± 0.22^a^	0.005
	Ash, %	14.16 ± 0.36	13.65 ± 0.35	14.03 ± 0.18	13.93 ± 0.32	0.707
	Carcass energy, Kcal/kg^2^	5881.9 ± 19.8	5899.6 ± 26.3	5901.8 ± 20.5	5949.2 ± 6.9	0.174
Biofloc composition	Dray matter (DM), %	23.81 ± 0.37^d^	26.22 ± 0.19^c^	28.00 ± 0.05^b^	29.63 ± 0.06^a^	0.001
	Crude protein (CP), %	31.00 ± 0.22^a^	29.50 ± 0.14^b^	27.80 ± 0.08^c^	26.55 ± 0.18^d^	0.001
	Ether extract (EE), %	6.06 ± 0.11^a^	5.22 ± 0.04^b^	4.70 ± 0.09^c^	3.89 ± 0.08^d^	0.001
	Ash, %	23.91 ± 0.15^d^	25.01 ± 0.18^c^	26.71 ± 0.23^b^	28.60 ± 0.25^a^	0.001
	NFE^1^	39.05 ± 0.03^b^	40.28 ± 0.01^a^	40.79 ± 0.40^a^	40.97 ± 0.36^a^	0.004
	Gross energy (GE), Kcal/kg^2^	3925.0 ± 3.8^a^	3811.0 ± 11.8^b^	3688.0 ± 3.6^c^	3548.0 ± 11.7^d^	0.001

Means within the same row with different superscripts are significantly different (*P* ≤ 0.05). Where BFT = biofloc; S0 = water with a salinity of 0 ppt (fresh water); S12 = water with a salinity of 12 ppt; S24 = water with a salinity of 24 ppt; and S36 = water with a salinity of 36 ppt; ^1^ NFE is nitrogen free extract = 100%-(protein + lipids + ash); ^2^ Gross energy (GE) was calculated as 5.64, 9.44, and 4.11 Kcal/g for protein, lipids, and carbohydrates, respectively (NRC 1993)

### Blood biochemical analyzes, immunity, and antioxidant activity

**Fig. 3:**
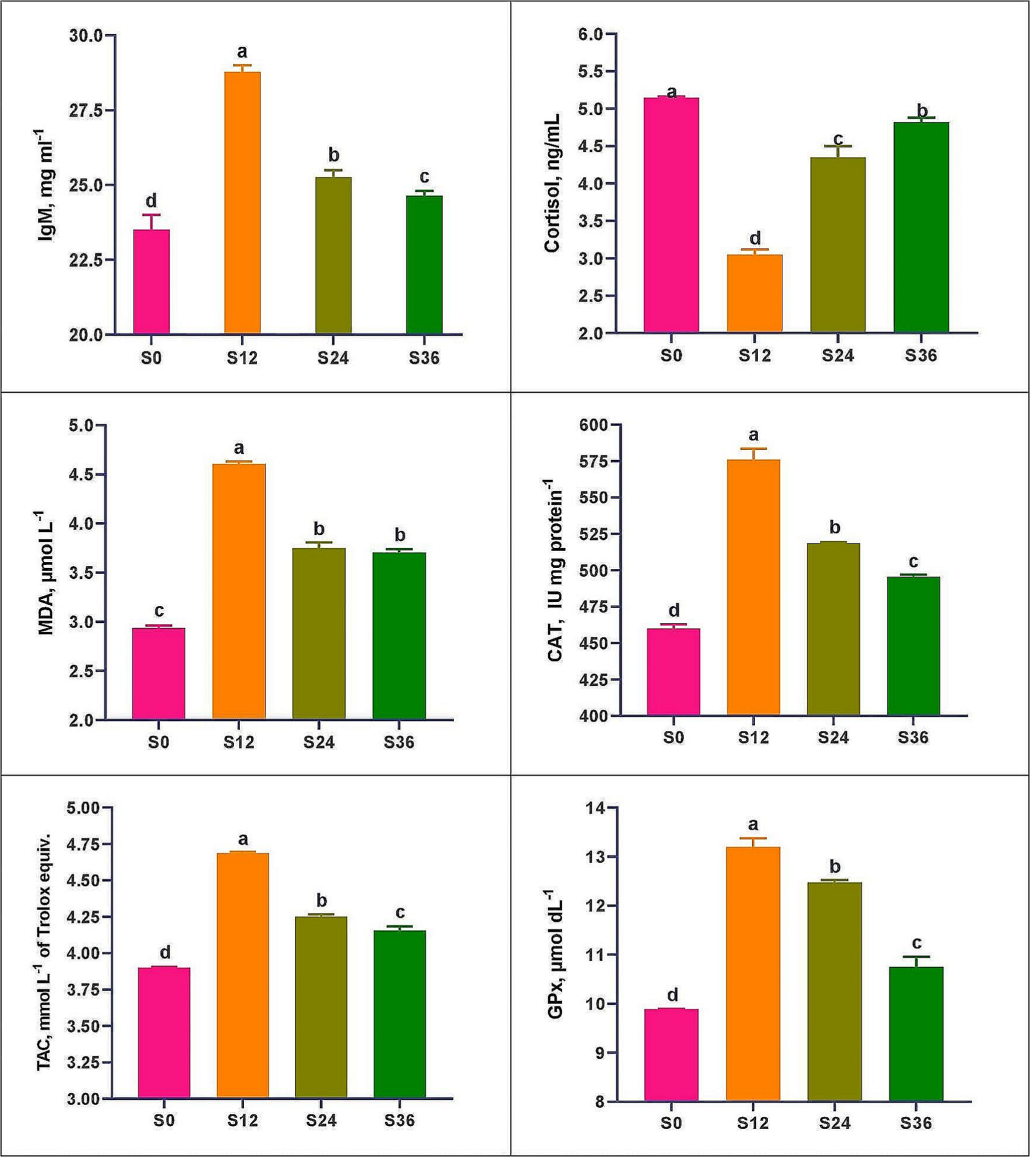
Serum immunity, stress, and antioxidant indices of Florida red tilapia grown in BFT-based tanks using underground water with different salinities, for 75 days. Where BFT = biofloc; S0 = water with a salinity of 0 ppt (fresh water); S12 = water with a salinity of 12 ppt; S24 = water with a salinity of 24 ppt; and S36 = water with a salinity of 36 ppt

Table 5 shows results for serum kidney parameters (urea, CRE, ammonia and uric acid) and liver (ALT, AST and ALP). In serum kidney parameters, the S12 group exhibited the lowest significant values (*P* < 0.05) compared to the other treatments, with CRE dropping to the lowest significant value at S24. Group S0 displayed the highest values. In the same context, liver enzyme (ALT) in the S12 group had the lowest significant levels compared to the other treatments. The ALT and ALP values decreased as salinity increased, with the lowest values occurring in the 36 ppt group. Figure 3 illustrates serum immunity, stress, and antioxidant indicators. Group S12 had the highest significant IgM values and the lowest COR levels compared to the other treatments, while S0 demonstrated the opposite. IgM levels were 22.5% higher in S12, while COR was 40.8% lower in S0.

**Table 5:** Serum liver enzymes and kidney function indicators of Florida red tilapia grown in BFT-based tanks using underground water with different salinities, for 75 days

Treatment		S0	S12	S24	S36	*P* value
Kidney parameters	Urea (mg/dL)	29.0 ± 0.58^a^	14.5 ± 0.29^c^	14.5 ± 0.29^c^	17.5 ± 0.29^b^	0.001
	Creatinine (mg/dL)	0.705 ± 0.01^a^	0.610 ± 0.02^b^	0.435 ± 0.00^c^	0.635 ± 0.01^b^	0.001
	Uric acid (mg/dL)	0.700 ± 0.02^a^	0.460 ± 0.01^d^	0.560 ± 0.01^c^	0.615 ± 0.00^b^	0.001
	Ammonia (mg/dL)	23.5 ± 0.29^a^	18.0 ± 0.58^c^	20.5 ± 0.29^b^	21.5 ± 0.29^b^	0.001
Liver enzymes	AST (U/l)	49.50 ± 0.87^a^	9.45 ± 0.09^d^	14.50 ± 0.87^c^	27.00 ± 0.58^b^	0.001
	ALT (U/l)	79.50 ± 1.44^a^	12.50 ± 0.23^b^	12.00 ± 0.58^b^	11.00 ± 0.58^b^	0.001
	ALP (mg/dl)	257.5 ± 2.02^a^	222.0 ± 1.15^b^	208.0 ± 0.58^c^	84.5 ± 0.29^d^	0.001

Values in the same column with a different superscript are significantly different (*P* ≤ 0.05). Where BFT = biofloc; S0 = water with a salinity of 0 ppt (fresh water); S12 = water with a salinity of 12 ppt; S24 = water with a salinity of 24 ppt; and S36 = water with a salinity of 36 ppt; AST = aspartate aminotransferase; ALT = alanine aminotransferase; ALP = alkaline phosphatase

The immunological parameters (TP, ALB, and GLO) are shown in Table 6. In general, the S12 group had significantly better TP and GLO records than the S0 and S36 groups, although the S12 and S24 groups had the highest significant ALB levels. The glucose results revealed that S12 had the highest significant values, while S0 had the lowest values. Figure 3 illustrates the antioxidant parameters (MDA, CAT, TAC, and GPx). The results showed that the antioxidant indices improved at higher salinity levels (12-36ppt), with the best values favoring the S12 group and the lowest in the S0 group. MDA was 56.9% higher, while CAT was 25.2% higher than in S0. GPx records were much further away in high salinity treatments (S12, S24, and S36) than at S0, as seen in Fig. 3. The serum lipid profile and digestive enzymes of Florida red tilapia are shown in Table 6. The results showed that the levels of CHO and TG were significantly (*P* < 0.05) higher in S12 and S24 than in S0 and S36. LDL had the lowest significant levels (10.00 ± 0.58) in the S12 group. S12 had the highest amylase values compared to the other treatments, while S24 had the highest lipase values.

**Table 6:** Serum immunity parameters, lipid profile and digestive enzymes of Florida red tilapia grown in BFT-based tanks using underground water with different salinities, for 75 days

Treatment	S0	S12	S24	S36	*P* value
Total protein (g/dl)	1.770 ± 0.01^d^	2.095 ± 0.01^a^	1.960 ± 0.01^b^	1.850 ± 0.02^c^	0.001
Albumin, A (g/dl)	0.56 ± 0.01^b^	0.61 ± 0.01^a^	0.62 ± 0.01^a^	0.43 ± 0.01^c^	0.001
Globulin, G (g/dl)	1.210 ± 0.01^d^	1.485 ± 0.00^a^	1.340 ± 0.01^c^	1.420 ± 0.01^b^	0.001
Glucose (mg/dl)	438.0 ± 1.15^d^	784.5 ± 2.02^a^	586.5 ± 4.91^b^	530.0 ± 8.66^c^	0.001
Cholesterol (mg/dl)	52.5 ± 0.87^c^	63.0 ± 0.58^a^	53.5 ± 0.87^c^	59.5 ± 0.29^b^	0.001
Triglyceride (mg/dl)	128.0 ± 0.58^c^	180.0 ± 0.58^a^	135.0 ± 0.58^b^	121.5 ± 0.29^d^	0.001
HDL (mg/dl)	15.5 ± 0.29^c^	18.0 ± 0.59^b^	13.5 ± 0.29^d^	19.5 ± 0.29^a^	0.001
LDL (mg/dl)	12.35 ± 0.38^b^	10.00 ± 0.58^c^	14.50 ± 0.29^a^	15.00 ± 0.00^a^	0.001
Amylase (U/L)	221.36 ± 7.14^c^	262.35 ± 2.11^a^	246.20 ± 0.46^b^	226.50 ± 0.87^c^	0.001
Lipase (U/L)	21.5 ± 0.29^b^	30.5 ± 0.87^a^	32.0 ± 1.15^a^	21.5 ± 0.87^b^	0.001

Values in the same column with a different superscript are significantly different (*p* ≤ 0.05). Where BFT = biofloc; S0 = water with a salinity of 0 ppt (fresh water); S12 = water with a salinity of 12 ppt; S24 = water with a salinity of 24 ppt; and S36 = water with a salinity of 36 ppt; HDL = High-density lipoprotein; LDL = low-density lipoprotein

### Histological changes in the liver and intestinal tract

**Fig. 4:**
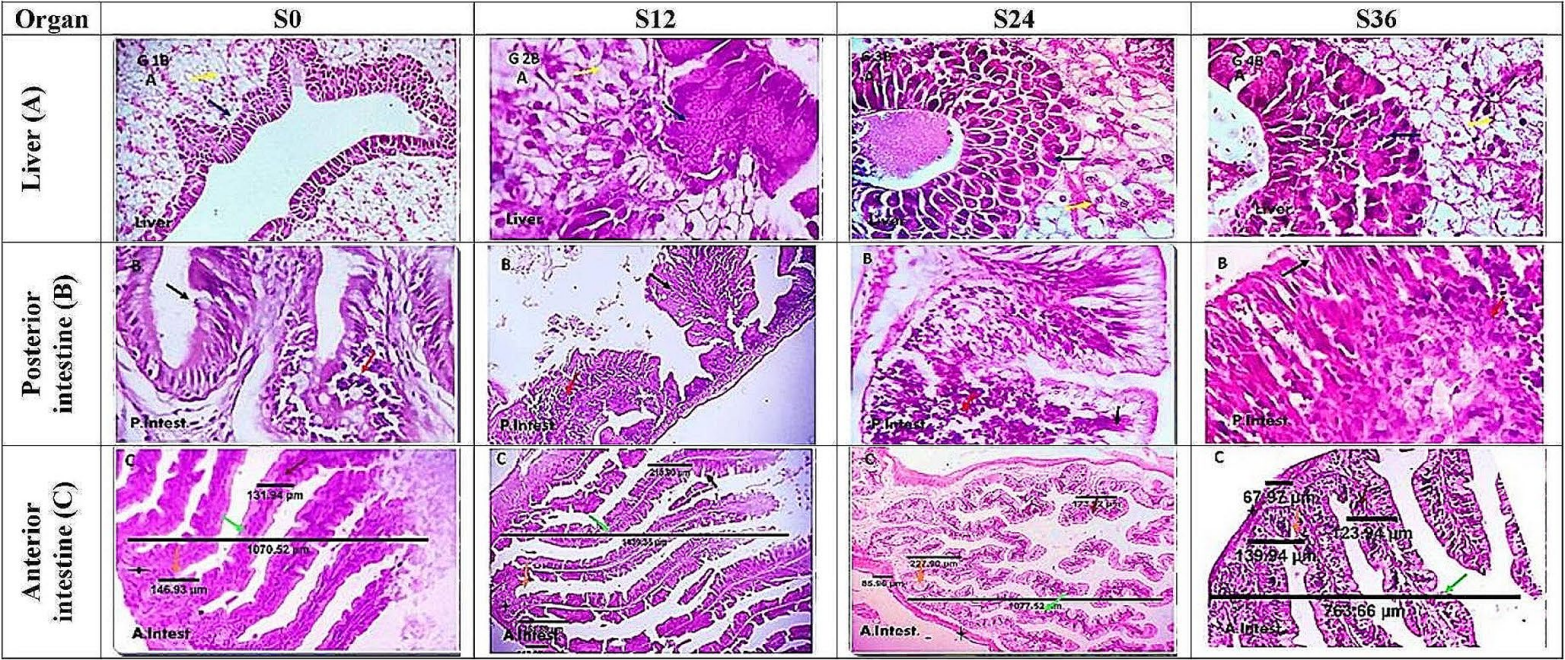
Liver (**A**), posterior (**B**) and anterior (C) intestines of Florida red tilapia grown in BFT-based tanks using underground water with different salinities, for 75 days. Where BFT = biofloc; S0 = water with a salinity of 0 ppt (fresh water); S12 = water with a salinity of 12 ppt; S24 = water with a salinity of 24 ppt; and S36 = water with a salinity of 36 ppt.  Fish showing apparently normal lobular arrangement, central venules, and portal area structures with close similarity to the mammalian hepatic portal structures but with the presence of intra-hepatic pancreases (dark blue arrows) enclosing a branch from the portal vein. The hepatocytes (yellow arrows), sinusoids and reticulio-endothelial system are well-formed and histologically normal.  The posterior intestine of all examined fish groups was nearly similar, however, a mild to moderate number of lymphocytes were seen infiltrating the lamina epithelial (red arrows), with a normal active histologic morphology and goblet cells population (black arrows). H&E X 100, 200 and 400.  The anterior intestine of the examined fish exhibited simple columnar epithelium, lamina propria, submucosa, tunica muscularis and serosa. The epithelial cells appeared as long and thin columnar cells and the mucosa showed surface microvilli. A variable number of goblet cells and infiltrating normal immune cells (lymphocytes) are shown (gray arrows). The dimensions of the intestinal villi (green arrows), crypts of Lieberkühn length (orange arrows) in addition to villous width (brown arrows) are seen

Table 7 shows the length and width of the intestinal villi, the length of the Lieberkühn crypts, and the thickness of the muscle coat. The length (1419.1 ± 15.3 μm) and the width (215.7 ± 1.75 μm) were 33.4 and 67.7% higher in the S12 group compared to the S0 group. The number of intestinal goblet cells in the interior section (#/HPF) appeared significantly (*P* < 0.05) highest in S24, followed by S12, while S0 and S36 did not reveal significant differences. Figure 4 illustrates the histomorphology of the liver (A), anterior (C, D), and posterior (B) intestines of FRT examined with various levels of salinity in the rearing water. Figure 4 shows detailed information, including more healthy outputs for fish raised in S12 and S24.

**Table 7:** Histology parameters of intestine (villous length, villous width, crypts of Lieberkühn length and muscle coat thickness) in Florida red tilapia grown in BFT-based tanks using underground water with different salinities, for 75 days

Treatment*	S0	S12	S24	S36	*P* value
Intestine villi length, µm	1064.2 ± 4.0^b^	1419.1 ± 15.3^a^	1071.9 ± 3.3^b^	760.8 ± 2.1^c^	0.001
Intestine villi width, µm	128.6 ± 1.76^c^	215.7 ± 1.75^a^	166.1 ± 4.18^b^	120.9 ± 1.58^c^	0.001
Crypts of Lieberkühn length, µm	144.7 ± 1.22^c^	248.8 ± 1.78^a^	223.6 ± 2.44^b^	135.7 ± 2.16^d^	0.001
Muscle coat thickness, µm	105.8 ± 1.33^b^	112.2 ± 0.90^a^	83.8 ± 1.22^c^	65.0 ± 1.90^d^	0.001
Intestine goblet cell, #/HPF	11.67 ± 0.88^c^	15.00 ± 0.58^b^	25.67 ± 0.33^a^	12.00 ± 1.00^c^	0.001
Total goblet cell number, #	12,419	21,287	27,517	9,130	-

Values in the same column with a different superscript are significantly different (*P* ≤ 0.05). Where BFT = biofloc; S0 = water with a salinity of 0 ppt (fresh water); S12 = water with a salinity of 12 ppt; S24 = water with a salinity of 24 ppt; and S36 = water with a salinity of 36 ppt

### Pathogenic bacterial load in the rearing water

Table 8 shows the average pathogenic bacterial load in the rearing water of the experimental tanks for FRT. The findings revealed significant variations (*P* < 0.05) in treatment outcomes in five of the six species tested. The S12 treatment had the largest significant number of *Vibrio sp* and *total aerobic count*, while the S24 treatments had the lowest. *Aeromonas sp.* was found to be significantly more prevalent in the S36 treatment than in the other treatments. *Streptococcus sp*. and *Staphylococcus aureus* were found in the largest significant amounts after S36 treatment. There were no significant differences among the treatments for *Salmonella sp*.

**Table 8:** Pathogenic bacterial load in culture water of Florida red tilapia grown in BFT-based tanks using underground water with different salinities, for 75 days

Treatments	S0	S12	S24	S36	*P* value
*Streptococcus sp.*	117.0 ± 1.73^c^	153.7 ± 1.86^b^	28.7 ± 0.88^d^	246.0 ± 2.31^a^	0.001
*Vibrio sp.*	10.7 ± 1.45^b^	49.7 ± 0.88^a^	1.0 ± 0.58^c^	9.0 ± 1.15^b^	0.001
*Salmonella sp.*	1.7 ± 0.88	2.0 ± 1.00	5.7 ± 4.26	0.7 ± 0.33	0.459
*Total aerobic count*	103.0 ± 1.73^c^	253.7 ± 2.33^a^	13.7 ± 0.88^d^	202.0 ± 1.15^b^	0.001
*Staphylococcus aureus*	20.7 ± 2.03^b^	10.3 ± 1.20^c^	4.7 ± 0.33^d^	49.0 ± 2.31^a^	0.001
*Aeromonas sp.*	1.0 ± 0.58^b^	1.3 ± 0.88^b^	1.7 ± 0.88^b^	13.0 ± 0.58^a^	0.001

Values in the same column with a different superscript are significantly different (*P* ≤ 0.05). Where BFT = biofloc; S0 = water with a salinity of 0 ppt (fresh water); S12 = water with a salinity of 12 ppt; S24 = water with a salinity of 24 ppt; and S36 = water with a salinity of 36 ppt

## Discussion

In this study, we investigated the feasibility of using desert groundwater with varying salinity levels (0, 12, 24, and 36 ppt) in a BFT system for the culture of FRT. Our results demonstrate that salinity significantly impacted water quality, fish performance, and physiological responses. In agreement with our findings, salinity levels influenced the same parameters along with carcass composition, chromatic deformity, and other characteristics of hybrid red tilapia not only under running water culture condition (García-Ulloa et al. 2001; Nassar et al. 2021), but also in BFT conditions (Osman et al. 2023). There is currently no research on the employment of USW and BFT technology in FRT culture. However, other traits of hybrid red tilapia (*O. mossambicus* × *O. niloticus*) have been examined using BFT (Banuelos-Vargas et al. 2021; Osman et al. 2023), and only one study has addressed USW and BFT using the same species (Kumari et al. 2021).

The current study found that using USW at various salinity levels had significant effects on all monitored water quality parameters (Table 2), with the most appealing effects observed in the TAN, NH_3_, nitrite, hardness and floc volume findings. The current findings are consistent with those of (Luo et al. 2017; Kumari et al. 2021; Osman et al. 2023) about the impact of salinity levels on water quality. Significant reduction in NH_3_ can be linked to the effective performance of the FRT at salinity levels of 12 and 24 ppt, resulting in reduced waste output and ultimately release of NH3 into the environment, compared to salinity of 0 and 36 ppm. This explanation agrees with the findings of Nassar et al. (2021) ) that salinity levels between 13 and 26 ppt are more suitable for FRT performance than 0 or 39 ppt, resulting in a lower ammonia concentration in rearing water. In addition to the poor performance of the previous hybrid with salinity increasing from 0 to 20 ppm (Kumari et al. 2021) and from 0 to 39 ppm (Osman et al. 2023) there were increases in TAN, nitrite, and nitrate. Furthermore, low and high salinity outside the optimal range can increase ammonia excretion by altering metabolic processes in fish organs, especially the liver (the main organ involved in ammonia production in fish) (Chew and Ip 2014), and the gills the main site of ammonia excretion in the surrounding water (Wright and Wood 2012). For example, when environmental factors such as salinity, temperature, pH, and oxygen in an aquarium become out of balance, the fish’s gills adjust their structure and size to accommodate these changes, and this may cause a metabolic imbalance that leads to high ammonia levels (Wilkie 2002; Wright and Wood 2012; Chew and Ip 2014). In addition, the low quality of BFT microorganisms that serve as natural food leads to an imbalance in water quality parameters in culture tanks. Regarding floc volume, it seems that water salinity had a significant effect on the BFT system, as found in this study and other studies (Kumari et al. 2021; Osman et al. 2023). The microbial community (structure and diversity) in a BFT system can change with salinity (decreases with higher salinity) and the presence of dominant phyla (*Bacteroidetes* and *Proteobacteria*, including nitrifying and denitrifying bacteria) (Dong et al. 2022) may be less adapted to saltwater than to freshwater. Zhang et al. (2023) discovered that microbial community structure and diversity decreased with salinity. Using activated sludge as an inoculum for high-salinity bioflocs can help solve this problem (Zhang et al. 2023). The current study found that increased salinity (12–36 ppt) had a beneficial influence on growth and feeding utilization compared to salinity 0 ppt, the best values occurring at 12 ppt. However, the regression equations for final weight and FCR accurately defined the optimum level at 20.0 ppt. These results are consistent with the findings of Kumari et al. (2021), who obtained the best results for red tilapia at 20 ppt. Also, Osman et al. (2023) had better results for final weight, gain, FCR, and PER at 18 ppt under biofloc culture conditions. The improved growth results and feeding utilization in groups S12 and S24, as well as the significant increase in the survival rate in group S12 compared to the other groups, can be attributed to the improvement in water quality, specifically TAN and NH_3_. Furthermore, the improvement in kidney function, liver enzyme activity, digestive enzyme activity, and intestinal histology of fish at 12 and 24 ppt strengthens this hypothesis. According to Kumari et al. (2021) a BFT system with increased salinity is excellent for the growth of this euryhaline species, as long as the water quality is maintained. The presence of heterotrophic microbial populations in the culture system degrades organic matter and nitrogenous compounds, improving water quality, and creating a healthy habitat for fish (Wang et al. 2019). Furthermore, Nassar et al. (2021) discovered that red tilapia exhibited the highest growth and feed utilization at 26 ppt in a running water culture system. The survival rate results of the current study were comparable, with a higher value of 12 ppt, validating the data on growth and feed efficiency. Ekasari and Maryam (2012) found that utilizing BFT improved the management of water quality and feed efficiency in red tilapia, whereas Haridas et al. (2017) found the same in the GIFT strain. Therefore, growing red tilapia in USW with a salinity of 20 ppt using the BFT culture system opens up a new era of desert marine aquaculture that is both economically profitable and environmentally beneficial. This reduces the dependence on freshwater, which can be used for other important activities such as agriculture.

In the current study, there were no statistically significant differences in carcass composition indicators of tilapia (dry matter, protein, lipids, ash and carcass energy) at salinity levels of 0–24 ppt, however, at 36 ppt, both protein and lipids differed significantly from the other salinities. This result may be explained by the fact that salinity 36 is above the optimal level for growth and osmoregulation. This hypothesis is somewhat consistent with the findings of Kumari et al. (2021), Nassar et al. (2021) and Osman et al. (2023) in red tilapia. Kumari et al. (2021) pointed out that when the salinity is appropriate for the physiological metabolism of this euryhaline species, more energy can be used for growth. Furthermore, the lower percentage of crude protein in fish flesh (group S36) can be related to the decrease in floc volume and protein content consumed by FRT, since biofloc microorganisms can provide highly digestible protein and contribute to increased body protein. In this study, the biofloc composition showed that as salinity increased, the dry matter, ash, and NFE content increased and the lipid and protein content decreased. Our findings are consistent with those of red tilapia raised in salinity levels (0 to 20 ppt) (Kumari et al. 2021) and (0, 18, 36 ppt) Osman et al. (2023) and red tilapia grown in seawater with biofloc plus probiotics Banuelos-Vargas et al. (2021). According to Tacon et al. (2002) microbial ash content measures the mineral and trace element acquisition capacity of *Litopenaeus vannamei* during flocculation and growth. The absence of large fluctuations (up and down) in the carcass composition indices of fish supports the commercial application of the results of the study and alleviates concerns about the quality of fish produced in the desert, particularly with ideal well water analyzes.

The biochemistry of fish plasma is affected, either directly or indirectly, by a number of biotic and abiotic variables, including water salinity (Abdel-Rahim et al. 2020). Serum kidney and liver parameters are critical in determining the health status and welfare of any aquatic organism (Abdel-Rahim et al. 2023). The COR hormone is released after acute stress as a primary rebuttal in fish, and changes in plasma cortisol levels depend on the nature of the stress stimuli and stress period, as well as the magnitude and strength of the stress (Ray and Sinha 2014). Inconstancy in plasma urea shows decreased liver function or impaired gill regulatory ability in conjunction with CRE (Zhou et al. 2009). ALP is a lysosomal enzyme involved in cellular phosphate metabolism (Javahery et al. 2019). ALT and AST are liver enzymes that participate in protein metabolism and gluconeogenesis (El Basuini et al. 2020; Ghafarifarsani et al. 2022). Elevated liver enzymes (ALP, ALT, AST) and/or kidney markers (urea, CRE, ammonia, uric acid) can be indicative of apoptosis, erythrocyte hemolysis, and liver or kidney disease due to pollution or stress (El-Kady et al. 2022). In our study, kidney parameters and liver enzymes had the lowest values at salinity levels 12 and 24 ppt, while higher values were found at salinity levels 0 and 36 ppt. The results are consistent with the findings of (Nassar et al. 2021; Faisal et al. 2023; Osman et al. 2023) using red tilapia. When kidney function is severely impaired, the kidney glomerular filtration process removes CRE from the blood, leading to elevated levels. Kulkarni and Pruthviraj (2016) found that fish exposed to high salinity showed a slight increase in serum CRE, indicating that there was no kidney dysfunction. In the current study, COR levels were lower at salinity levels of 12–24 ppt and higher at salinities of 0 and 36 ppt. This result is consistent with the findings of Ruiz-Jarabo et al. (2019) for meagre. The previous authors discovered that fish adapted to high salinity (39 ppt) can consume more energy metabolites than those adapted to 12 ppt. This hormone mobilizes energy metabolites, increasing carbohydrate consumption and hepatic lipid mobilization, which is the primary source of lipid deposits (Chatzifotis et al. 2010).

Serum TP is a significant clinical indicator of stress, health, humoral defense system, and the welfare of aquatic organisms (El-Kady et al. 2022; Abdel-Rahim et al. 2023). Additionally, TP is vital to maintain blood pH and osmotic pressure from ecological pressures, which can decrease plasma protein values (Abdel-Rahim et al. 2020). ALB and GLO are two crucial components of TP and the main source of immunoglobulins production (Ahmadifar et al. 2019). ALB manages lipid transport and overall metabolism (Ghafarifarsani et al. 2022). The IgM family is the considerable dominant immunoglobulin (Ig) in fish serum that protects against viruses and pathogens. In this Ig-mediated humoral defense, activation of the complement system occurs, viruses and toxins are counteracted, and pathogens are eliminated by phagocytes (El-Kady et al. 2022). In our study, plasma immunological indices in fish exhibited higher values at salinity levels 12–24 ppt and lower values for salinities 0 and 36 ppt. FRT maintained at 12–24 salinity showed an enhanced immune state, as demonstrated by increased serum TP in the present study. Abdel‐Rahim et al. (2020) for meagre and Laiz‐Carrión et al. (2005) for gilthead seabream under varying salinity conditions are in agreement with this result. The serum TP level can be decreased by environmental stresses such as hyper or hypo-salinity, as well as by diseases (Louis et al. 2003). Decreased TP values at 0 ppt and 36 ppt salinity can be due to loss of protein in osmolality (Abdel‐Rahim et al. 2020), and/or decreased fish appetite, reducing synthesis and absorption capacity, or increasing protein loss by haemodilution (Patriche et al. 2011). Kumari et al. (2021) discovered that salinity had a beneficial effect on the innate immune system of red tilapia, making them less susceptible to disease when grown under salinity conditions of 20 ppt. TG plays an important role in the metabolism, storage, and provision of primitive cellular energy and its evaluation reflects nutritional status and lipid metabolism (El Basuini et al. 2020). CHO is an essential structural component of the biomembrane, the outer layer of blood lipoproteins, and precursors of steroid hormones (Ghafarifarsani et al. 2022). The increased levels of TG and CHO in the S12 group may be indicators of the appropriate environment in which the fish were grown. In addition, this may indicate the welfare of the fish and that the lower stressors in the rearing water allowed excess energy to be stored rather than expended to resist stress.

The nonenzymatic and enzymatic antioxidant defense systems of fish represent protection against oxidative stress and oxidative damage in tissues (Shahin et al. 2023). The antioxidant system primarily composed of SOD, CAT and GPx plays an important role in maintaining fish health and protecting living cells from free radicals, excessive inflammation, and apoptosis. The three most important first-line antioxidant enzymes that detoxify superoxide ions and hydrogen peroxide are SOD, CAT, and GPx (Yousefi et al. 2022; Elhetawy et al. 2023b). Fluctuations in water salinity can cause oxidative stress and reactive oxygen species (ROS) in aquatic organisms (Lushchak 2011). At increased salinity levels (12-36ppt), antioxidant defense response indices (CAT, GPx and TAC) improved, with the S12 group having the highest values and the S0 group the lowest. The results are consistent with those of Banuelos-Vargas et al. (2021) and (Kumari et al. 2021) for red tilapia. Increased antioxidant enzyme activity indicates cellular activation of antioxidant defense and protection against ROS (Elhetawy et al. 2023b). Poor environmental conditions, such as excessive stocking density and heavy metal pollution, can reduce CAT and GPX activity in fish (Banuelos-Vargas et al. 2021; Elhetawy et al. 2023b). The presence of microorganisms in BFT can contribute to improving the innate immunity and antioxidant defense system of farmed fish (Mansour and Esteban 2017). This may be due to microbial components, unknown growth factors, or even some probiotic microorganisms such as Bacillus and Lactobacillus present in biofloc (Zhao et al. 2016). Furthermore, immune responses are known to be modulated in various ways by nutrients such as proteins, lipids, antioxidants, vitamins, carotenoids, and minerals (Mansour and Esteban 2017; Abdel-Rahim et al. 2024). The digestion of biofloc in the intestine can increase the quantity and/or quality of these nutrients and possibly stimulate fish cell defenses in the form of phagocytosis (Xu and Pan 2014) or respiratory burst. Furthermore, the additional protein source of biofloc is rich in key amino acids (Ekasari et al. 2014) and may also contribute to immune function and antioxidant defense in BFT cultured fish (Li et al. 2009). In the present study, digestive enzyme levels were higher at salinity levels of 12–24 ppt and lower at salinity levels of 0 and 36 ppt. In line with the findings of the present study Mozanzadeh et al. (2021), found that the activities of total protease and lipase increased in the *Acanthopagrus latus* and *Lates calcarifer* intestine with increasing water salinity up to 35 and 24, respectively, before decreasing at higher water salinity. Amylase can promote metabolic activity, glucose handling in the blood and increase liver glycogen, leading to increased starch utilization and glucose metabolism in fish (Elhetawy et al. 2024). Furthermore, lipase can enhance fish metabolic activity, intestinal immunity, and fish meat properties (Liang et al. 2022). The histological results of the fish intestines in the current study revealed the same conclusion. Moutou et al. (2004) found that gilthead sea bream raised at 20‰ salinity were less active in pancreatic proteases than those reared at 33‰ salinity.

Histopathological changes in the liver and intestine provide insight into structural modifications when organisms adapt to different salinity levels. Improved intestinal health (villi length and width) and increased numbers of goblet cells in S12 and S24 fish may be due to the increased production of endogenous enzymes that stimulate cholecystokinin secretion and exocrine pancreatic secretion, thus adjusting the gastrointestinal physiology and stimulating digestion and absorption of food and nutritional complements (Ghafarifarsani et al. 2022; Elhetawy et al. 2024). This improvement can be attributed to the positive effect of biofloc, which acts as a probiotic to enhance the goblet cells of the villi and the extra lateral branches of the villous epithelium, which improves the performance and health of the fish (Zhao et al. 2016). These histological results of the intestinal tract confirmed the growth and feed utilization data from the current study with no damage seen in all groups. This is consistent with Osman et al. (2023), who found that red tilapia had a normal villi structure and that intestinal tissue was not affected by salinity variation, and comparable to those published by Tran-Ngoc et al. (2017). This study showed that FRT had healthier livers in all salinity groups. The histomorphological structure of the liver showed that fish have normal lobular arrangement, central venules and portal area structures, well-formed hepatocytes, sinusoids, and the reticulio-endothelial system. On the contrary, Faisal et al. (2023) reported that histological examination of the red tilapia gills and livers revealed two types of liver neoplasms, significant damage, vascular congestion, telangiectasis, and round cell infiltration in the gills at high and low salinity levels. In our experience, the lack of such damage in FRT liver and intestinal tissue may be due to the positive role that the biofloc microbiota plays in maintaining tissue health in fish. Despite its lack of primary osmoregulatory function, the liver plays a crucial role in the metabolism of glycogen and glucose (de Azevedo et al. 2015; do Vale Figueiredo et al. 2022).

Biofloc-based systems have been found to include a much higher concentration of microbes, including both pathogenic, nonpathogenic, and beneficial microorganisms (Liu et al. 2019). The presence of pathogenic bacteria in culture water is an additional indicator of water quality and can explain certain symptoms or findings that would be difficult to interpret without the presence of these specific bacteria. In the current study, increasing the number of *Vibrio sp*. at salinity 12 ppt and subsequently reducing it to salinity 24 ppt could be related to the fact that the optimal salinity range for the growth of *Vibrio sp.* is at brackish water, whereas salinity 24 ppt is somehow outside of the range. This explanation is consistent with prior research, which found that the optimal salinity range for the growth of *Vibrio sp.* is near fresh (1 ppt) to brackish (17 ppt) (Louis et al. 2003). *V. vulnificus* grows best at 5–25 ppt (Kaspar and Tamplin 1993). However, the increased total number of *Vibrio sp*. at 36 ppt may be due to previous findings in this study that water quality indicators and fish performance deteriorated at 36 ppt compared to previous salinity (12–24 ppt). This is consistent with the findings of Sullivan and Neigel (2018) who revealed that higher salinities can increase stress levels in blue crabs, which in turn can increase infection rates and more than offset a minor negative effect on the development of *V. cholerae*. Also, previous authors stated that the stressful effect of increased salinities on blue crabs could be a secondary mechanism that significantly promotes infection by *V. cholerae.* The above hypothesis may be a logical explanation for the similar increase in the total number of *Aeromonas sp., Streptococcus sp., Staphylococcus aureus* and *Total aerobic count* at 36 ppt salinity after a decrease at 24 ppt salinity.

In a commercial BFT system, *Vibrio alginoticus*, *Vibrio harveyi*, *Vibrio rotiferianus*, *Photobacterium sp., and Photobacterium damselae* were identified (Abakari et al. 2021). However, BFT aquaculture systems are highly biosecured, with low *Vibrio sp*. densities in *L. vannamei* culture. This is due to bioflocs with high levels of poly-β-hydroxybutyric (PHB), which protects animals from infection. PBH is an important bioactive component that represents the most dominant polymer in biofloc (Supono et al. 2014; Klanian et al. 2020), and has been linked with positive impacts such as the potency to prohibit pathogens in the intestinal tract, on aquatic organisms (Gao et al. 2019). The idea of this biopolymer is based on the degradation of short-chain fatty acids (SCFAs), and SCFAs which are known compounds with antimicrobial properties (Klanian et al. 2020; Laranja and Bossier 2020). PHB has been used to improve growth performance and FCR, as well as to provide resistance against pathogenic infection and ammonia stress in post-larval shrimp farming (Gao et al. 2019). Heterotrophic bacteria compete for food and niches, reducing *Vibrio* incidence (Halet et al. 2007). Furthermore, the presence of *Aeromonas sp*. in this study is anticipated, despite the fact that the number of bacteria discovered remains low. This result is consistent with those of Gou et al. (2019) and Pérez-Fuentes et al. (2018). Bioflocs play a crucial role in the biosecurity and sustainability of the aquaculture system, but they still face pathogenic infestations and outbreaks of diseases. In this regard, Pérez‐Fuentes et al. (2018) discovered seventeen different species of pathogenic bacteria in water, as well as fifteen species of other bacteria in the intestines of tilapias raised in BFT and fed various feed diets, including *Aeromonas sp. and Vibrio sp*. Further research is needed to elucidate the mechanisms by which salinity influences the composition of the biofloc microbial community and its impact on pathogen abundance.

## Conclusions

This study explored the feasibility of using USW for sustainable aquaculture in desert regions. Given the high salinity tolerance of FRT and the water-saving properties of BFT, this study investigated the suitability of USW for FRT culture in BFT systems. The results demonstrate that USW with a salinity of 12–24 ppt is effective for FRT aquaculture, promoting growth performance, reducing toxic ammonia levels, and improving the physiological health of internal organs. In particular, a salinity of around 20 ppt appears to be optimal for these benefits. These findings suggest that desert regions can use USW for sustainable aquaculture by implementing BFT systems with FRT, thus mitigating the environmental impact of fish farming and making productive use of marginal water resources.

## Electronic supplementary material

Below is the link to the electronic supplementary material.

Supplementary Material 1

## Data Availability

No datasets were generated or analysed during the current study.

## Declarations

### Animal welfare statement

The University of Alexandria Institutional Animal Care and Use Committee accepted the study (IACUC approval AU19/23/10/28/3/33). The authors confirm that all techniques were performed in accordance with the relevant guidelines and regulations and that the study is reported in accordance with the ARRIVE guidelines.
