# Supplementary material for: Enhancing Florida red tilapia aquaculture: biofloc optimization improves water quality, pathogen bacterial control, fish health, immune response, and organ histopathology across varied groundwater salinities
Source: Vet Res Commun. 2024 Jul 3;48(5):2989–3006. doi: 10.1007/s11259-024-10433-w (PMC11457711; doi:10.1007/s11259-024-10433-w)
Supplement: Supplementary file 1 — Supplementary Material 1 [file 11259_2024_10433_MOESM1_ESM.docx]

**2.9. Pathogenic bacterial load**

Sampling, preparing, and counting the bacterial isolates:

Water samples were collected for bacterial counting of the experimental units using previously described methods (PHE, 2014). The glass bottles containing samples must be kept in the dark at a constant low temperature. Water samples were collected in 500 mL sterile screw-caped bottles, as previously described by Austin (1988). To culture, 1 ml of each sample was diluted in 99 ml of saline and cultivated using the pour plate technique (PPT) on nutrient agar plate medium at 30 °C for 18-24 hours. The numbers of examined bacteria were calculated as CFU/100 mL. The bacterial examination followed ISO 9308/1 (1990b) and 7899/2 (1984) membrane filtering techniques. Diluted samples (1 ml) were filtered using a 0.45 µm pore with a 47 mm diameter and a sterile cellulose membrane grid. Faecal streptococci membranes were placed on Slanetz and Bartley media after 72 hours of incubation at 37°C. The dark red colonies were counted to detect *Vibrio sp.* The membranes were placed on thiosulphate citrate bile salt sucrose (TCBS) agar and incubated at 37°C for 24 hours. Large green and/or yellow colonies were considered to be Vibrio sp. Salmonella sp. were detected and counted using bismuth sulphite agar medium and Salmonella-Shigella (SS) agar. Black colonies were counted after 18-24 hours of incubation at 35-37 °C. *Aeromonas sp.* were detected using an *Aeromonas* isolation medium basis. After 18-24 hours of incubation at 35-37 °C, dark green opaque colonies with dark centers were counted. To detect and count *Staphylococcus aureus*, membranes were put onto mannitol salt agar and incubated at 35 °C for 18-24 hours. Yellow colonies were then counted. To detect and count *Pseudomonas sp.,* membranes were placed onto *Pseudomonas* isolation agar. Blue-green colonies were found after incubation for 40-48 hours at 35°C.

Media:

The media used in this work are described below. The composition is expressed in g/L. The pH of the media was adjusted to 7.5 before sterilization. The autoclave ran for 15 minutes at 121°C Celsius.

1. Nutrient agar medium (Atlas and Bartha, 1997) composed of: yeast extract, 2; beef extract, 1; peptone, 5; sodium chloride, 5. Agar (15-20) was added for obtaining nutrient agar medium.
2. Slanetz and Bartley media (Slanetz, and Bartley, 1957) composed of Tryptose 20, Yeast Extract 5, Dextrose 2, Dipotassium Phosphate 4, Sodium Azide 0.4, Agar 10, 2,3,5Triphenyl Tetrazolium Chloride 0.1. Final pH at 25°C 7.2 ± 0.2.
3. Mannitol salt agar (Abo-Elela and Farag, 2004) used for isolating *Staphylococcus* spp*.*: Peptone complex, 10; beef extract, 1; sodium chloride, 75; mannitol 10; phenol red, 0.025 and agar 15.
4. Thiosulfate citrate bile salt sucrose agar (TCBS) (Kobayashi *et al.,* 1963) used for isolating *Vibrio* spp.: Yeast extract, 5; peptone, 10; sodium thiosulfate, 10; sod-citrate, 10; Ox bile, 8; sucrose, 20; sodium chloride, 10; ferric chloride, 1; Bromothymol blue, 0.04; thymol blue, 0.04 and agar, 14.
5. Aeromonas agar (Atlas and Bartha, 1997) for the isolation of *Aeromonas sp*. it is composed of: Proteose peptone; 5.0, yeast extract; 3.0, L. Lysine monohydrochloride; 3.5, L. Arginine monohydrochloride; 2.0, Sorbitol; 3.0, Inositol; 2.5, Lactose; 1.5, Xylose; 3.75, bile salts #3; 3.0, sodium thiosulfate; 10.67, sodium chloride; 5.0, Ferric ammonium citrate; 0.8, Bromothymol blue; 0.04, Thymol blue; 0.04 and agar; 12.5.
6. Salmonella Shigella Agar (SS Agar) (Atlas and Bartha, 1997) used to isolate Salmonella and Shigella. It is composed of: beef extract; 5.0, polypeptone; 5.0, lactose; 10.0, sodium citrate; 8.5, ferric citrate 10.0, sodium thiosulfate; 8.5, bile salts #3; 8.5, Neutral Red; 0.025, agar; 13.5 and Brilliant Green, 0.330mg.

**3. Results:**

*3.1. Water quality data and floc volume*

The following parameters were recorded: temperature (27.5 to 27.77 ◦C), pH (7.49 to 8.3), dissolved oxygen (6.53 to 6.88 ppm), total alkalinity (66.0 to 328.2 ppm), NH_3_ (15.13 to 31.67 ppb), nitrate-N (1.57 to 4.07 ppm), nitrite-N (0.31 to 0.46 ppm), and total hardness (67.67 to 5823.67 ppm) (Table 2 a). With the exception of temperature, all investigated parameters exhibited significant differences (P ≤ 0.05) among treatments. S12 had the lowest TAN and NH_3_ values, whereas S0 had the highest (Figure 1). Floc volume (mL/L) differed significantly (P ≤ 0.05) across treatments throughout the trial, with the highest volume (36.33) observed in S0 and the lowest (23.20) in S36 (Table 2 a). Table 2 b (Supplementary Information) displays the ranges of water quality parameters within each group over the 75-day experimental period.

**Table 2 b.** Physicochemical parameter of the rearing water of Florida red tilapia reared in a desert BFT culture system using underground water with different salinities during the experimental period.

| Parameters | S0 | S12 | S24 | S36 | *P* value |
| --- | --- | --- | --- | --- | --- |
| Temperature, ^◦^C | 27.50 (25.66- 28.43) | 27.73 (26.04- 28.74) | 27.67 (25.85- 28.74) | 27.70 (26.13- 28.63) | 0.434 |
| pH | 7.49 (7.09- 7.65)**^d^** | 7.63 (7.21- 7.87)**^c^** | 7.78 (7.27- 8.05)**^b^** | 8.03 (7.60- 8.29)**^a^** | 0.001 |
| DO, ppm | 6.88 (6.48- 7.13)**^a^** | 6.53 (6.18- 6.75)**^b^** | 6.58 (6.13- 6.87)**^b^** | 6.58 (6.15- 6.82)**^b^** | 0.001 |
| TAN, ppm | 0.486 (0.448-0.510)**^a^** | 0.232 (0.212- 0.244)**^d^** | 0.284 (0.258- 0.305)**^c^** | 0.356 (0.327- 0.374)**^b^** | 0.001 |
| NH3, ppb | 31.67 (29.23- 33.27)**^a^** | 15.13 (13.82- 15.97)**^d^** | 18.50 (16.64- 19.88)**^c^** | 23.23 (21.34- 24.41)**^b^** | 0.001 |
| Nitrate-N, ppm | 1.57 (1.30- 1.96)**^d^** | 2.57 (2.35- 2.88)**^c^** | 3.47 (3.20- 3.61)**^b^** | 4.07 (3.90-4.30)**^a^** | 0.001 |
| Nitrite-N, ppm | 0.310 (0.290-0.330)**^b^** | 0.337 (0.320-0.371)**^b^** | 0.350 (0.329-0.381)**^b^** | 0.460 (0.440-0.490)**^a^** | 0.001 |
| Alkalinity, ppm | 66.0 (61.4-69.3)**^d^** | 116.5 (104.1-127.6)**^c^** | 212.2 (191.9-225.4)**^b^** | 328.2 (302.1-344.8)**^a^** | 0.001 |
| Hardness, ppm | 67.7 (60.0-71.4)**^d^** | 2723.7 (2527-2837)**^c^** | 3895.3 (3689-4013)**^b^** | 5823.7 (5456-6021)**^a^** | 0.001 |
| Floc volume, ml/L | 36.33 (33.7-38.1)**^a^** | 30.40 (29.2-31.5)**^b^** | 27.73(26.7-28.4)**^c^** | 23.20 (22.0-24.2)**^d^** | 0.001 |

Treatments: (S0) salinity level 0 ppt; (S12) salinity level 12 ppt; (S24) salinity level 24 ppt; and (S36) salinity level 36 ppt; Values are average (the lowest and highest values). Values in the same row with a different superscript are significantly different (*P* ≤ 0.05)

**References:**

Abo-Elela GM, Farag AM (2004) Bacteriological quality and metal contents of *Diplodus vulgarus* and *Siganus rivulotus* in the Eastern Harbour water: a comparative study of freshly harvested and market fish. *Egyptian J. Aquatic Res.* 30(A): 216-225.

Atlas RM, Bartha R (1997) Microbial ecology fundamentals and applications. Benjamin/Cummings Science Publishing, Menlo Park, Calif.

Austin B (1988) The marine environment. In: Marine Microbiology, 111. Cambridge: Cambridge University Press. 1-11.

Kobayashi T, Enomato S, Sakazaki R, Kuwahara S (1963). A new selective medium for pathogenic *Vibrios*. TCBS agar (modified Nakanishi's agar). *Japanese*. *J. Bacteriol.* 18: 387-391.

PHE (2014) Public Health England. Preparation of samples and dilutions, plating and subculture. In Microbiology Services Food, Water and Environmental Microbiology. Standard Method FNES26 (F2), London: Public Health England. pp. 12–13.

Slanetz, LW, Bartley C H (1957) Numbers of enterococci in water, sewage, and feces determined by the membrane filter technique with an improved medium. *Journal of bacteriology*, *74*(5), 591–595. https://doi.org/10.1128/jb.74.5.591-595.1957
